# Supplementary material for: Causal Relationship between Aging and Anorexia Nervosa: A White-Matter-Microstructure-Mediated Mendelian Randomization Analysis
Source: Biomedicines. 2024 Aug 16;12(8):1874. doi: 10.3390/biomedicines12081874 (PMC11351342; doi:10.3390/biomedicines12081874)
Supplement: Supplementary file 1 [file biomedicines-12-01874-s001.zip › Supplementary Figure/Figure S4/Singe SNP plot of MR analysis for TL to ubm-b-1583.pdf]

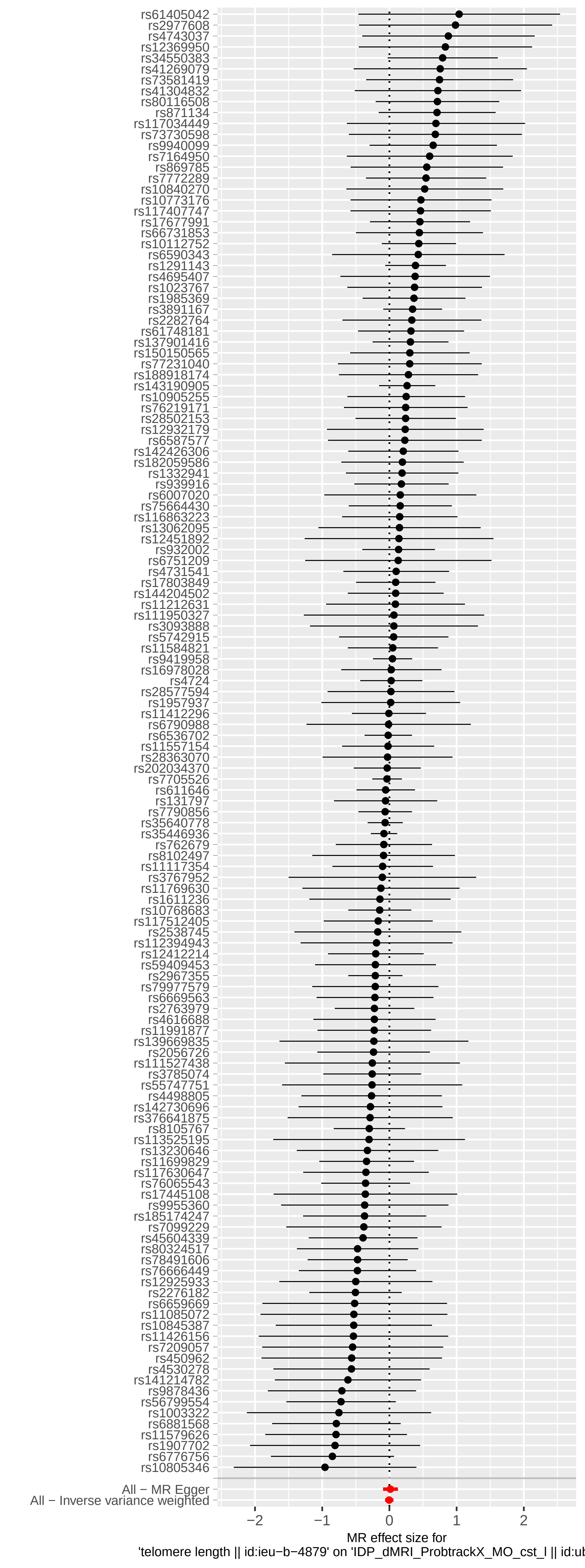
rs61405042
rs2977608
rs4743037
rs12369950
rs34550383
rs41269079
rs73581419
rs41304832
rs80116508
rs871134
rs117034449
rs73730598
rs9940099
rs7164950
rs869785
rs7772289
rs10840270
rs10773176
rs117407747
rs17677991
rs66731853
rs10112752
rs6590343
rs1291143
rs4695407
rs1023767
rs1985369
rs3891167
rs2282764
rs61748181
rs137901416
rs150150565
rs77231040
rs188918174
rs143190905
rs10905255
rs76219171
rs28502153
rs12932179
rs6587577
rs142426306
rs182059586
rs1332941
rs939916
rs6007020
rs75664430
rs116863223
rs13062095
rs12451892
rs932002
rs6751209
rs4731541
rs17803849
rs144204502
rs11212631
rs111950327
rs3093888
rs5742915
rs11584821
rs9419958
rs16978028
rs4724
rs28577594
rs1957937
rs11412296
rs6790988
rs6536702
rs11557154
rs28363070
rs202034370
rs7705526
rs611646
rs131797
rs7790856
rs35640778
rs35446936
rs762679
rs8102497
rs11117354
rs3767952
rs11769630
rs1611236
rs10768683
rs117512405
rs2538745
rs112394943
rs12412214
rs59409453
rs2967355
rs79977579
rs6669563
rs2763979
rs4616688
rs11991877
rs139669835
rs2056726
rs111527438
rs3785074
rs55747751
rs4498805
rs142730696
rs376641875
rs8105767
rs113525195
rs13230646
rs11699829
rs117630647
rs76065543
rs17445108
rs9955360
rs185174247
rs7099229
rs45604339
rs80324517
rs78491606
rs76666449
rs12925933
rs2276182
rs6659669
rs11085072
rs10845387
rs11426156
rs7209057
rs450962
rs4530278
rs141214782
rs9878436
rs56799554
rs1003322
rs6881568
rs11579626
rs1907702
rs6776756
rs10805346
All − MR Egger
All − Inverse variance weighted
−2
−1
0
1
2
MR effect size for
'telomere length || id:ieu−b−4879' on 'IDP_dMRI_ProbtrackX_MO_cst_l || id:ub
